# Identification of *Staphylococcus aureus*: DNase and Mannitol salt agar improve the efficiency of the tube coagulase test

**DOI:** 10.1186/1476-0711-9-23

**Published:** 2010-08-13

**Authors:** David P Kateete, Cyrus N Kimani, Fred A Katabazi, Alfred Okeng, Moses S Okee, Ann Nanteza, Moses L Joloba, Florence C Najjuka

**Affiliations:** 1 Department of Medical Microbiology, School of Biomedical Sciences, Makerere University College of Health Sciences, Upper Mulago hill road, Kampala, Uganda; 2 Department of Veterinary Parasitology and Microbiology, Faculty of Veterinary Medicine, Makerere University, Kampala, Uganda; 3 Current Address: P.O. BOX 25085, 00603, Nairobi, Kenya

## Abstract

**Background:**

The ideal identification of *Staphylococcus aureus *clinical isolates requires a battery of tests and this is costly in resource limited settings. In many developing countries, the tube coagulase test is usually confirmatory for *S. aureus *and is routinely done using either human or sheep plasma. This study evaluated Mannitol salt agar and the deoxyribonuclease (DNase) test for improving the efficiency of the tube coagulase test in resource limited settings. The efficiency of human and sheep plasma with tube coagulase tests was also evaluated.

**Methods:**

One hundred and eighty Gram positive, Catalase positive cocci occurring in pairs, short chains or clusters were subjected to growth on Mannitol salt agar, deoxyribonuclease and tube coagulase tests. Of these, isolates that were positive for at least two of the three tests (n = 60) were used to evaluate the performance of the tube coagulase test for identification of *S. aureus*, using PCR-amplification of the *nuc *gene as a gold standard.

**Results:**

Human plasma was more sensitive than sheep plasma for the tube coagulase test (sensitivity of 91% vs. 81% respectively), but both plasmas had very low specificity (11% and 7% respectively). The sensitivity and specificity of the tube coagulase test (human plasma) was markedly improved when Mannitol salt agar and DNase were introduced as a tri-combination test for routine identification of *Staphylococcus aureus *(100% specificity and 75% sensitivity). The specificity and sensitivity of Mannitol salt agar/DNase/tube coagulase (sheep plasma) combination was 100% and 67%, respectively.

**Conclusion:**

The efficiency of the tube coagulase test can be markedly improved by sequel testing of the isolates with Mannitol salt agar, DNase and Tube coagulase. There is no single phenotypic test (including tube coagulase) that can guarantee reliable results in the identification of *Staphylococcus aureus*.

## Background

*Staphylococcus aureus *is a ubiquitous commensal bacterium on human skins and anterior nares, but frequently causes severe infections in humans [1]. Rapid and direct identification of *S. aureus *is crucial for proper management of patients with skin infections, abscesses, septicemia/bacteremia, gastroenteritis, endocarditis, toxic shock syndrome and certain food intoxications [2,3]. In developing countries, phenotypic tests are the mainstay in the diagnosis of staphylococcal infections, in which coagulase tests are usually confirmatory for *S. aureus *[4-8]. Coagulase testing is performed using the slide coagulase (SCT) or the tube coagulase (TCT) methods [9]. Although these tests efficiently identify *S. aureus*, their performances vary from setting to setting and need improvement [3,6].

Several laboratories in developing countries screen for presumptive *Staphylococcus aureus *based on growth on Mannitol salt agar (MSA) and/or DNase tests and confirmation is done with the TCT. In many settings, the use of the TCT is curtailed by reliance on human plasma, since the recommended plasmas (from rabbit, horse [10]) are either expensive or if locally available, are of poor quality. Human plasma is reported to give discordant results [6]; usually obtained from blood banks as outdated materials, it contains variable amounts of CRF (Coagulase-Reacting Factor) and anti-staphylococcal antibodies [9]. This type of plasma is not recommended for coagulase tests [9]. Other factors which make human plasma inappropriate for the coagulase tests include; a high burden of viral infections (such as HIV/AIDS, Hepatitis B and C) in resource limited settings that can render the plasma risky to laboratory workers and prior to use, it must be screened for safety. There are also ethical issues accompanying the use of human specimens. Conversely, the efficiency of other plasmas in many laboratory settings varies and has to be determined for proper identification of *S. aureus *with coagulase tests.

Although coagulase tests are invaluable for identification of *Staphylococcus aureus*, few studies have evaluated their use in routine practice [10]. In addition, diagnostic laboratories are occasionally faced with organisms with biochemical characteristics that do not fit into the patterns of a known genus and species [11]. Furthermore, there are some problems associated with coagulase tests; firstly, some human CoNS (Coagulase Negative Staphylococci) produce clumping factor and may be falsely positive with the SCT [9,12]. Secondly, some staphylococci of animal origin are clumping factor negative and tube coagulase positive; these may be misidentified as *S. aureus*, unless the fermentation of Mannitol is utilized in addition [9]. Whereas Mannitol salt agar was developed for the presumptive isolation of *S. aureus *in a single step, which is convenient for diagnostic laboratories, Mannitol positive CoNS have been reported in nasal and clinical specimens from Nigeria and Japan [13,14]. Furthermore, Mannitol negative MRSA (Methicilin Resistant *Staphylococcus aureus*) was reported from clinical specimens in Kwazulu Natal province, South Africa [15]. In view of the above, the common identification methods for *S. aureus *were evaluated, aiming at improving the diagnosis of *S. aureus *through a combination of available phenotypic methods.

This study reveals that there is no single test (including the tube coagulase test) that can guarantee reliable results for the identification of *S. aureus*. However, improved diagnostic sensitivity and specificity of the tube coagulase test were achieved upon simultaneous testing of the isolates with DNase and Mannital salt agar. In order to improve the identification of *S. aureus *in resource limited settings, sequel testing of the isolates with Mannitol salt agar, tube DNase and coagulase is proposed.

## Methods

### Study setting

This study was done in the Clinical Microbiology and Molecular Biology laboratories of the Department of Medical Microbiology, Makerere University College of Health Sciences, from January through June, 2009. It was a laboratory based study that involved frozen clinical isolates from patients' blood, cerebral spinal fluid, anterior nares, skin and wound swabs.

### Inclusion/exclusion criteria

One hundred and eighty Gram positive, Catalase positive cocci occurring in pairs, short chains or clusters were subjected to growth on MSA, tube coagulase and DNase tests (see below). Of these (N = 180), isolates that were positive for at least two of the three tests (n = 60) were used to evaluate the performance of the tube coagulase test for identification of *S. aureus*, using PCR-amplification of the *nuc *gene (which is specific for *S. aureus*) as the gold standard.

### Phenotypic identification of *Staphylococcus aureus*

Isolates were incubated at 37°C for 24 hours on blood agar and then sub-cultured on TS (Tryptic Soya, Liofilchem, Italy) agar (Fisher, Leicestershire, UK). Single colonies were tested with tube coagulase and DNase test and growth on MSA. To evaluate the performance of the individual tests or a combination of tests, sequel testing of the isolates was done beginning with MSA, followed by DNase and TCT.

To confirm fermentation of mannitol, growth of yellow colonies on MSA (Oxoid, Cambridge, UK) surrounded by yellow zones after 24 hours of incubation at 37°C indicated a positive result. DNase test was performed by incubating the isolates for 24 hours at 37°C on DNase agar (Scharlau, Barcelona, Spain), and pouring an excess (~15 ml) of 1 N HCl. Excess acid was removed with a vacuum pipette and clear zones around the bacterial colonies indicated DNase positive colonies. For tube coagulase tests, colonies of test isolates were re-suspended in 2 ml of citrated sheep or human plasma in sterile glass test-tubes. Since citrate is utilized by enterococci [16], pure colonies of Gram positive, Catalase positive staphylococci (catalase tests preceded coagulase reactions) were used. Positive control tubes with citrated plasma and coagulase producing strain of *S. aureus *ATCC 25923 (which efficiently coagulates citrated plasma) were included. To rule out citrate utilization by other microorganisms, control TCTs containing citrated plasma with *Staphylococcus epidermidis *ATCC 12228 were included. In addition, negative control tubes containing citrated plasma alone (with no cultures inoculated) were included. The tubes were incubated at 35°C for 4 hours and observed for clot formation. Where clotting did not occur, the tubes were incubated at room temperature for an additional 18 hours [9]. Tubes were studied without agitation in order not to disrupt partially formed clots.

### Molecular assays

PCR-amplification of the *nuc *gene was used as a baseline test. Reaction mixes were done in a Cleanspot ultraviolet workstation (COV Lab products, Michigan, USA). The reactions contained 20 pmoles each of the *nuc *forward and reverse primers (5'-GCGATTGATGGTGATACGGTT-3' and 5'-AGCCAAGCCTTGACGAACTAAAGC-3', respectively, synthesized by Eurofins-MWG-Operon, Ebersberg, Germany), 1.5 units of *Taq *DNA polymerase (Thermo-Fisher, Surry, UK) and 1 μl of custom PCR master mix (ThermoFisher, Surry, UK). In the post-amplification room, ~100 ng of staphylococcal chromosomal DNA was added to the reactions as template. Amplifications were done in a Peltier thermocycler (MJ Research, Waterman, MA, USA) under the following conditions: initial denaturation at 94°C, 5 min, followed by 37 cycles each consisting of a denaturation at 94°C, 1 min; primer annealing at 55°C, 0.5 min, and extension at 72°C, 1 min; followed by a final extension at 72°C, 7 minutes. After this, the amplicons were mixed with 5 μl of DNA loading buffer and electrophoresed in a 1% agarose gel in TAE buffer (Tris, acetate and EDTA). Control reactions included templates of *Staphylococcus aureus *ATCC 25923 (positive control), *Staphylococcus epidermidis *ATCC 12228 and nuclease free water (negative controls).

### Quality control

To minimize cross contamination, standard microbiological procedures were strictly followed. Positive and negative controls were always included in the test reactions. DNA extraction and PCR-amplifications were done in molecular laboratories that are separate from the clinical microbiology laboratory where cultures were grown. The PCR laboratory has designated sections for pre-amplification, amplification and post-amplification, with a unidirectional movement of staff.

### Data analysis

The data were analyzed using a 2 × 2 contingency table for diagnostic specificity and sensitivity (table 1). Diagnostic sensitivities and specificities were calculated as follows:

**Table 1:** Deriving Sensitivity, Specificity, Positive/Negative Predictive Values for the common identification tests for *S. aureus*

Test results	*Staphylococcus aureus*	Other staphylococci	Total
Positive	a	b	a + b
Negative	c	d	c + d
Total	a + c	b + d	(a + b) + (c + d) = n

Where,

a = True positives

b = False positives

c = False negatives

d = True negatives

Diagnostic sensitivity = d/(b + d)

Diagnostic specificity = a/(a + c)

Positive predictive value (PPV) = a/(a + b)

Negative predictive value (NPV) = d/(d + c)

n = 60

Sensitivity (%) = [True positive/(True Positive + False Positive)] × 100

Specificity (%) = [True Negative/(True Negative + False Negative)] × 100

The positive predictive value (PPV) (%) = [True Positive/(True Positive + False Positive)] × 100

The negative predictive value (NPV) (%) = [True Negative/(True Negative + False Negative)] × 100.

## Results and discussion

Coagulase testing is the single most reliable method for identifying *Staphylococcus aureus *[9]. Coagulase production can be detected using either the slide coagulase test (SCT) or the tube coagulase test (TCT). Slide coagulase detects bound coagulase (also called "clumping factor") [9], which reacts directly with fibrinogen in plasma, causing rapid cell agglutination. Negative isolates following SCT require confirmation with the superior TCT, since strains deficient in clumping factor usually produce free coagulase. Tube coagulase detects secreted extracellular free coagulase that reacts with a substance in plasma called "Coagulase-Reacting Factor" (CRF) to form a complex, which in turn reacts with fibrinogen to form fibrin (the clot) [9]. Strains of coagulase-positive-animal staphylococci have been isolated from human clinical specimens, yet some animal staphylococcal isolates also ferment mannitol [13]. This study evaluated the performance of TCTs, DNase and MSA, the phenotypic methods commonly used in the identification of *Staphylococcus aureus*.

### Detection of *Staphylococcus aureus *with the available phenotypic tests

PCR-amplification of the *nuc *gene, which was used as a baseline test, detected 32 *Staphylococcus aureus *of the 60 staphylococcal isolates, see table 2. Of the 32 *nuc*-positive *Staphylococcus aureus *isolates, MSA detected the highest number of isolates (30 of 32, 94%) while the TCT (human and sheep plasma respectively) detected 29 and 27 of the 32 isolates (91% and 84% respectively), table 2. DNase detected the least number of isolates (24 of 32, 75%).

**Table 2:** Identification of *S. aureus *with the common tests in comparison to PCR-detection of the *nuc *gene

Other tests		*nuc *PCR		
	
	Outcome	Positive (% of 32)	Negative (% of 28)	Subtotal
MSA	Positive	30 (94)	6 (21)	36
	Negative	2 (6)	22 (79)	24
DNase	Positive	24 (75)	1 (4)	25
	Negative	8 (25)	27 (96)	35
**^1^**Human plasma	Positive	29 (91)	25 (89)	54
	Negative	3 (9)	3 (11)	6
Human plasma/MSA	Positive	27 (84)	6 (21)	31
	Negative	0	3 (11)	3
Human plasma/DNase	Positive	21 (60)	1 (4)	22
	Negative	0	3 (11)	3
Human plasma/MSA/DNase	Positive	21 (60)	1 (4)	22
	Negative	0	3 (11)	3
**^2^**Sheep plasma	Positive	26 (81)	26 (93)	52
	Negative	6 (19)	2 (8)	8
Sheep plasma/MSA	Positive	24 (75)	6 (21)	30
	Negative	0	2 (8)	2
Sheep plasma/DNase	Positive	18 (56)	1 (4)	19
	Negative	0	1 (4)	1
Sheep plasma/MSA/DNase	Positive	18 (56)	1 (4)	19
	Negative	0	2 (8)	2
MSA/DNase	Positive	24 (74)	1 (4)	25
	Negative	2 (6)	22 (79)	24

**^1^**: tube coagulase (human plasma)

**^2^**: tube coagulase (sheep plasma)

MSA: Mannitol salt agar

DNase: Deoxyribonuclease

PCR: Polymerase Chain Reaction

Nine of the 32 *nuc*-positive *Staphylococcus aureus *isolates (28%) were coagulase negative, implying that some isolates may be misidentified by the TCT as a sole test. The coagulase negative *Staphylococcus aureus *may probably be MRSA isolates which are reported to react weakly or negatively with TCTs [17], or rare *Staphylococcus aureus *strains that are reported to be coagulase negative [9]. Two *Staphylococcus aureus *isolates (6%) were also MSA negative. Shittu *et al *also reported mannitol negative *Staphylococcus aureus *that was methicillin resistant [15]. Furthermore, eight of the 32 *nuc*-positive *Staphylococcus aureus *isolates were DNase negative (25%). Rao *et al *reported DNase negative *Staphylococcus aureus *but with no explanation for these findings [18]. Six staphylococci other than the *nuc*-confirmed *Staphylococcus aureus *isolates produced yellow colonies on MSA, and similar findings were reported by other investigators [19-24]. Another rare finding in this study was an isolate that was DNase positive but MSA-negative and tube coagulase-negative (i.e. non-*Staphylococcus aureus*). We presume this isolate could have been *Staphylococcus schleiferi *subsp. *coagulans*, which according to the National Standard Method is also DNase positive [9].

### Single phenotypic tests are inefficient for the identification of *Staphylococcus aureus*

We used sensitivity and specificity to evaluate the performance of individual tests in detecting *Staphylococcus aureus*. Growth on MSA was the most sensitive (94% sensitivity), followed by the TCT (human and sheep plasma, 91% and 81% sensitivity respectively) while the DNase test was the least sensitive (75% sensitivity). Conversely, the DNase test was the most specific (96% specificity) followed by MSA (79% specificity), while the TCT (human plasma and sheep plasma) was the least specific (11% and 7%, respectively), table 3. Overall, of the individual tests studied, growth on MSA was the best at identifying *Staphylococcus aureus *(94% sensitivity and 79% specificity). Our results slightly differ from those of Han *et al*, who reported sensitivity and specificity of 76.5% and 99.6%, respectively, for MSA [25]. D'Souza *et al*, also reported sensitivity of 71%, a little lower than ours [26]. The high sensitivity of MSA in detecting *Staphylococcus aureus *could be due to its ability to isolate pathogenic *Staphylococcus aureus *[27], differentiating coagulase negative staphylococci from coagulase positive staphylococci.

**Table 3:** Sensitivity, Specificity, Negative/Positive Predictive values for the common diagnostic tests for clinical *S. aureus*

Test	Sensitivity (%)	Specificity (%)	NPV (%)	PPV (%)
MSA	94	79	92	83
DNase	75	96	77	96
**^1^**Human plasma	91	11	33	54
**^2^**Sheep plasma	81	7	25	50
MSA/DNase	96	92	92	96
Human plasma/MSA	33	100	100	82
Human plasma/DNase	75	100	100	95
Human plasma/DNase/MSA	75	100	100	94
Sheep plasma/MSA	25	100	100	80
Sheep plasma/DNase	50	100	100	95
Sheep plasma/MSA/DNase	67	100	100	95

**^1^**: Tube coagulase (human plasma)

**^2^**: Tube coagulase (sheep plasma)

MSA: Mannitol salt agar

DNase: Deoxyribonuclease

PCR: Polymerase Chain Reaction

For tube coagulase, human plasma was more sensitive than sheep plasma (91% vs. 81% sensitivity), implying that using sheep plasma with TCTs may detect more false negative isolates. Thus, in this setting, it is unlikely that sheep plasma will replace human plasma for routine TCTs. Our data is in agreement with previous findings in which sensitivity of 94-100% was reported by other investigators [28-30]. However, in this study, the sensitivity for human and sheep plasma was higher than what was reported in other settings, where values as low as 54.5% (human plasma) and 45.5% (sheep plasma) were reported [28]. Therefore, with coagulase tests, plasma performance varies with setting and the choice of plasma can influence the efficiency of the test. Also, the use of EDTA or citrate as anticoagulant influences the performance of the test. Noteworthy, citrate only affects coagulase reactions of enterococci where it is utilized [16], but does not affect coagulase producing organisms such as *Staphylococcus aureus *[16].

The DNase test had a sensitivity of 75% and a specificity of 96%, which are comparable to other studies in which a sensitivity of 75% and a specificity of 100% were reported [31]. In this study, the DNase test was the most specific of all tests and had the least number of false positive isolates (there was only one false positive). This was in agreement with other reports, in which the DNase test was reported as superior to the TCT [28,31]. Growth on MSA was also highly specific (79% specificity), while the least specific test was the tube coagulase (human and sheep plasma, 11% and 7% specificity, respectively).

In contrast, other studies reported high specificity (93.6%) for human and sheep plasma with TCTs [32]. The low specificity in our study may partly be due to the non specific detection of other coagulase positive staphylococci, such as *Staphylococcus schleiferi *subspecies *coagulans*, *Staphylococcus delphini, Staphylococcus intermedius *and *Staphylococcus hyicu*s. *Staphylococcus delphini *and *Staphylococcus intermedius *are rare clinical isolates while *Staphylococcus hyicu*s is indeterminate (with prevalence ranging from 11% to 89% [17]). The prevalence of these isolates may be high in certain settings. Although MSA and DNase had high specificities and sensitivities, as individual tests, their use in routine identification of *Staphylococcus aureus *is curtailed by their ability to detect other bacterial isolates [14], and are mostly used during initial screens [6]. Indeed, Mannitol salt positive CoNS (*Staphylococcus caprae, S. hemolyticus *and *S. saprophyticus*) have been reported in Nigeria and Japan [13,14]. Thus, in certain settings, if used individually to identify *Staphylococcus aureus*, common phenotypic tests may be insufficient; some isolates will be misidentified, either as *Staphylococcus aureus *or CoNS.

### A combination of MSA and DNase improves the tube coagulase test

We then evaluated the sensitivity and specificity of test combinations (i.e. MSA/DNase/TCT) at detecting *Staphylococcus aureus*, aiming at improving the performance of the TCT. We achieved absolute specificity (100%) in detecting *Staphylococcus aureus *with test combinations, with the exception of the DNase/MSA combination (92% specificity). Conversely, the sensitivity of test combinations varied, with the MSA/DNase/TCT (human plasma) being the most sensitive (75% sensitivity), while MSA/DNase/TCT (sheep plasma) was the least sensitive (25% sensitivity), table 3.

A combination of MSA/DNase resulted in specificity and sensitivity of 92% and 96%, respectively, and this would be the combination of choice for identification of *Staphylococcus aureus*. However, since both tests are not specific to *Staphylococcus aureus *and can detect other bacterial isolates, the dual combination can only be used to improve the TCT. Although other test combinations-MSA/DNase/TCT (human plasma) and MSA/TCT (human plasma) had specificity of 100%, they had varying sensitivity (75% and 33%, respectively). In this line, a combination of MSA/DNase/TCT (human plasma) is better at identifying *Staphylococcus aureus *(100% specificity, 75% sensitivity) than the MSA/TCT (human plasma) combination (100% specificity, 33% sensitivity). Likewise, the DNase/TCT (sheep plasma) and MSA/TCT (sheep plasma) and MSA/DNase/TCT (sheep plasma) combinations had an absolute specificity (100%) but with varying sensitivity (50%, 25% and 67%, respectively).

Thus, the efficiency of the tube coagulase can be improved through simultaneous testing that includes both DNase and MSA. For higher sensitivity and specificity, sequel identification of *Staphylococcus aureus *may commence with MSA, DNase and then TCT. Noteworthy, the improved specificity of the TCT did not significantly alter the initially observed high sensitivity. The use of MSA prior to TCTs/DNase is highly recommended due to the clumping factor negative and tube coagulase positive staphylococci that are increasingly being recovered from human infections. These isolates also produce a heat stable DNase and can be misidentified as *Staphylococcus aureus*. However, these strains can be differentiated from *Staphylococcus aureus *by their failure to produce acid from maltose, lactose and mannitol. Furthermore, rare strains of *Staphylococcus aureus *can be coagulase negative [9], yet animal isolates (*S. intermidius, S. hyicus, S. delphini *and *S. schleiferi *subsp. *coagulans*) can be tube coagulase positive [9,33,34]; differentiation of these also requires growth on MSA.

### Predictive values

Growth on MSA had the highest negative predictive value (NPV) followed by DNase, while tube coagulase had the lowest NPV (which did not match the high sensitivity initially observed, see table 3). Conversely, test combinations gave absolute NPVs (100%) with the exception of DNase/MSA (NPV of 92%), table 3. The high NPVs of the test combinations, particularly those involving the TCT reveal that test combinations can be reliably used in routine identification of *Staphylococcus aureus*. DNase had the highest positive predictive value (PPV), followed by MSA (96% and 83%, respectively). Conversely, tube coagulase alone resulted in average PPVs (54% and 50%), see table 3. Interestingly, test combinations resulted in high PPVs, with those involving DNase having the highest PPVs (95%), see table 3. Thus, as for NPVs, the specificity of the TCT can be improved by a simultaneous identification of the isolates with DNase and MSA.

Therefore, the ideal identification of *Staphylococcus aureus *clinical isolates requires a battery of tests. *Staphylococcus aureus *infections are more frequent than those by other bacteria, particularly in settings with high HIV/AIDS prevalence [35-37]. This warrants correct identification of the isolates to achieve better treatment outcomes. Since options for improving the sensitivity and specificity are presented, these data will improve on the identification of *Staphylococcal aureus *in clinical specimens.

### Recommendations

For routine identification of *Staphylococcus aureus *from clinical specimens, we recommend simultaneous use of all the three tests (beginning with growth on MSA, DNase and Tube coagulase) in settings where rabbit or horse plasmas are not readily available. Screening of plasmas from other species (such as rabbit, goat, pig, and bovine), which can be cheaper and safer, and reported to be more efficient than human plasma with TCTs, is recommended. In addition, genotyping of clinical staphylococcal isolates is recommended to speciate CoNS isolates, and to determine the prevalence of rare *Staphylococcus aureus *and animal isolates in human specimens in Uganda.

## Conclusions

We have evaluated the performance of common laboratory tests used routinely in the identification of *Staphylococcus aureus *infections in Uganda. The identification of clinical *Staphylococcus aureus *still largely relies on the tube coagulase test, but it requires screening of the isolates with two additional tests (MSA and DNase) prior to TCTs, for improved efficiency. There is no single phenotypic test (including the tube coagulase test) that can guarantee reliable results in the identification of *Staphylococcus aureus*.

The specificity of sheep plasma was relatively low (even in combination with MSA/DNase), and may not be appropriate for the TCT in some settings. Thus, where rabbit or horse plasmas are unavailable, sheep plasma may not replace human plasma. Since human plasma is inappropriate or risky in some settings, the performance of plasmas from other animal species should be investigated for a replacement of human plasma in TCTs.

## Abbreviations

ATCC: American Type Culture Collection; CoNS: Coagulase Negative Staphylococci; CRF: Coagulase Reacting Factor; DNase: Deoxyribonuclease; EDTA: Ethylenediaminetetraacetic acid; HCl: Hydrochloric acid; HIV/AIDS: Human immunodeficiency virus/Acquired immunodeficiency syndrome; LB: Luria-Bertani; MRSA: Methicillin Resistant *Staphylococcus aureus*; MSA: Mannital salt agar; *nuc*: thermonuclease gene; PCR: polymerase chain reaction; PPV: Positive Predictive Value; NPV: Negative Predictive Value; STC: Slide Coagulase Test; TCT: Tube Coagulase Test; TS: Tryptic soy; TAE: Tris Acetate-EDTA

## Competing interests

The authors declare that they have no competing interests.

## Authors' contributions

FCN conceived and supervised the study. DPK wrote the manuscript and co-supervised the study. CNK performed the experiments in partial fulfillment for his BSC degree. The other authors provided intellectual support to CNK and contributed to the writing of the manuscript. All authors read and approved the final manuscript.
